# Clinical and Biomarker Changes in Premanifest Huntington Disease Show Trial Feasibility: A Decade of the PREDICT-HD Study

**DOI:** 10.3389/fnagi.2014.00078

**Published:** 2014-04-22

**Authors:** Jane S. Paulsen, Jeffrey D. Long, Hans J. Johnson, Elizabeth H. Aylward, Christopher A. Ross, Janet K. Williams, Martha A. Nance, Cheryl J. Erwin, Holly J. Westervelt, Deborah L. Harrington, H. Jeremy Bockholt, Ying Zhang, Elizabeth A. McCusker, Edmond M. Chiu, Peter K. Panegyres, PREDICT-HD Investigators and Coordinators of the Huntington Study Group

**Affiliations:** ^1^Department of Psychiatry, Carver College of Medicine, The University of Iowa, Iowa City, IA, USA; ^2^Department of Neurology, Carver College of Medicine, The University of Iowa, Iowa City, IA, USA; ^3^Department of Psychology, The University of Iowa, Iowa City, IA, USA; ^4^Department of Biostatistics, College of Public Health, The University of Iowa, Iowa City, IA, USA; ^5^Department of Biomedical Engineering, College of Engineering, The University of Iowa, Iowa City, IA, USA; ^6^Center for Integrative Brain Research, Seattle Children’s Research Institute, Seattle, WA, USA; ^7^Division of Neurobiology, Johns Hopkins University, Baltimore, MD, USA; ^8^College of Nursing, The University of Iowa, Iowa City, IA, USA; ^9^Department of Neurology, College of Medicine, The University of Minnesota, Minneapolis, MN, USA; ^10^Center of Excellence for Ethics, Humanities and Spirituality, Texas Tech University Health Sciences Center School of Medicine, Lubbock, TX, USA; ^11^Department of Psychiatry and Human Behavior, Division of Biology and Medicine, Brown University, Providence, RI, USA; ^12^Department of Radiology, School of Medicine, University of California, San Diego, CA, USA; ^13^Veterans Affairs San Diego Healthcare System, San Diego, CA, USA; ^14^Advanced Biomedical Informatics Group, LLC, Iowa City, IA, USA; ^15^Department of Biostatistics, Indiana University Fairbanks School of Public Health and Indiana University School of Medicine, Indianapolis, IN, USA; ^16^Department of Neurology, Westmead Hospital, The University of Sydney, Sydney, NSW, Australia; ^17^Department of Psychiatry, The University of Melbourne, Melbourne, VIC, Australia; ^18^Neurodegenerative Disorders Research Pty Ltd., Perth, WA, Australia

**Keywords:** Huntington disease, neurodegenerative disorders, premanifest, natural history, clinical trials, outcome measures, PREDICT-HD

## Abstract

There is growing consensus that intervention and treatment of Huntington disease (HD) should occur at the earliest stage possible. Various early-intervention methods for this fatal neurodegenerative disease have been identified, but preventive clinical trials for HD are limited by a lack of knowledge of the natural history of the disease and a dearth of appropriate outcome measures. Objectives of the current study are to document the natural history of premanifest HD progression in the largest cohort ever studied and to develop a battery of imaging and clinical markers of premanifest HD progression that can be used as outcome measures in preventive clinical trials. Neurobiological predictors of Huntington’s disease is a 32-site, international, observational study of premanifest HD, with annual examination of 1013 participants with premanifest HD and 301 gene-expansion negative controls between 2001 and 2012. Findings document 39 variables representing imaging, motor, cognitive, functional, and psychiatric domains, showing different rates of decline between premanifest HD and controls. Required sample size and models of premanifest HD are presented to inform future design of clinical and preclinical research. Preventive clinical trials in premanifest HD with participants who have a medium or high probability of motor onset are calculated to be as resource-effective as those conducted in diagnosed HD and could interrupt disease 7–12 years earlier. Methods and measures for preventive clinical trials in premanifest HD more than a dozen years from motor onset are also feasible. These findings represent the most thorough documentation of a clinical battery for experimental therapeutics in stages of premanifest HD, the time period for which effective intervention may provide the most positive possible outcome for patients and their families affected by this devastating disease.

## Introduction

Since 2001, Neurobiological Predictors of Huntington’s Disease (PREDICT-HD; NS040068) has examined early indicators of disease in over 1300 participants at risk for Huntington disease (HD). Previous publications documented motor, cognitive, psychiatric, and imaging correlates of emerging disease (Paulsen et al., 2006, 2008; Duff et al., 2007; Biglan et al., 2009). The discovery that changes due to HD begin many years prior to the onset of diagnosable HD has fostered growing consensus that intervention at the earliest possible phase is desirable. Experimental pharmacologic interventions are currently being tested and methods to silence the polyglutamine gene expansion are underway (Zhang et al., 2009; Ross and Tabrizi, 2011). Significant limitations to preventive clinical trials for HD include a lack of knowledge of the natural history of the disease and a dearth of outcome measures sensitive to disease changes in the earliest, or premanifest, stages of the disease.

To date, longitudinal studies have documented changes for 12, 24, and 36 months in smaller samples of premanifest HD (Tabrizi et al., 2013). Changes have been demonstrated in basal ganglia brain volumes (Aylward et al., 2011; Majid et al., 2011) and declines in cognitive performance, primarily emphasizing speeded cognitive processing (Kirkwood et al., 1999; Paulsen et al., 2001, 2013; Lemiere et al., 2002; Maroof et al., 2011), executive function (Snowden et al., 2002; Rupp et al., 2010; O’Rourke et al., 2011; Papp et al., 2013), working memory (Lemiere et al., 2004), visuomotor control (Hart et al., 2011; Tabrizi et al., 2012, 2013), and time production (Rowe et al., 2010). To our knowledge, only one other study has examined multiple phenotypic and biologic markers of change over time together in premanifest HD (Tabrizi et al., 2012, 2013). Conclusions from this group suggest imaging measures are useful for indexing change, but “decline in cognitive, quantitative motor, or oculomotor measures were limited at this stage” (Tabrizi et al., 2012).

The aims of the study are twofold. First, we aim to document the natural history of premanifest HD progression in the largest cohort ever studied. Second, we aim to develop a battery of imaging and clinical markers of premanifest disease progression. Findings are interpreted in terms of their utilitarian value for preventive clinical trials. Outcome measures are scaled to facilitate comparisons, and hypothesized effect sizes are used to determine sample sizes for randomized clinical trials (RCTs). Finally, graphical analysis is used to illustrate the course of premanifest HD.

## Materials and Methods

### Participants

Data in this study were collected from September 2001 to August 2012 from *N* = 1314 PREDICT-HD participants (1013 with premanifest HD and 301 controls) at 32 worldwide sites. All participants had completed genetic testing for HD prior to (and independent from) study enrollment. Participants with >35 CAG expansion repeats in the *HTT* gene were cases and those with repeats <36 served as gene-mutation negative comparison participants (controls). Exclusion criteria included other central nervous system disease, injury, or developmental disorder, or evidence of an unstable medical or psychiatric illness. The research protocol was approved by each site’s respective institutional review board and ethics committee, and all participants gave written informed consent and were treated in accordance with ethical standards.

Average years in the study (median) were six, with a range of 1–10. Over 75% of the sample had more than 3 years of data collected, 15% had 2 years, and <10% had 1 year. A subset of *N* = 204 gene-expanded participants received a motor diagnosis during the study (referred to as “converters”). Dropout was less than 5% per year. Sample size variation was due to a number of historic study design events: 1) the National Institutes of Health (NIH) grant that funded the study was renewed three times and participants were recruited for the duration of each individual grant; and 2) grant reviewers increased sample size on each renewal, so the total sample size increased as the length of possible study duration decreased.

### Premanifest staging groups: CAG-age product

Premanifest stages were based on a formula using genetic and demographic information to estimate proximity to HD diagnosis. The CAG-Age Product (CAP) score, computed as CAP_E_ = (age at entry) × (CAG − 33.66) (Zhang et al., 2011), was derived from an accelerated failure time (AFT) model predicting motor diagnosis from age at entry, CAG length, and their interaction. CAP_E_ is similar to the “disease burden” score of Penney et al. (1997) and presumably indexes the cumulative toxicity of mutant huntingtin. CAP_E_ can also be used to estimate the 5-year probability of motor diagnosis. Cutoffs for groups were CAP_E_ < 290 (Low), 290 ≤ CAP_E_ ≤ 368 (Medium), and CAP_E_ > 368 (High). The estimated time to diagnosis was, respectively, >12.78, 7.59–12.78, and <7.59 years. A dynamic (time-varying) CAP score was also used, denoted as CAP_D_, and computed with current age (rather than age at entry). CAP_D_ can be interpreted as a type of CAG-adjusted age metric (Ross et al., 2014). Table 1 shows descriptive statistics for demographic variables by premanifest groups defined at study entry (i.e., based on CAP_E_).

**Table 1:** **Demographic variables for progression groups**.

	Control^a^	Low	Medium	High
*N*	301	283	358	372
Female	194 (64.45%)	190 (67.14%)	235 (65.64%)	218 (58.60%)
Age	44.36 (11.41)	34.98 (7.92)	41.67 (9.56)	44.93 (10.09)
Education	14.87 (2.56)	14.57 (2.44)	14.54 (2.61)	14.33 (27.5)
Years in study	4.39 (2.29)	4.41 (2.68)	4.64 (2.61)	4.98 (2.52)
CAG	20.27 (3.49)	40.91 (1.62)	42.02 (2.04)	43.58 (2.74)
CAP_E_	NA	243.97 (34.55)	330.50 (23.05)	423.19 (51.25)
Converters	NA	11 (3.89%)	49 (13.69%)	144 (38.71%)

*^a^Percentage is based on group total. CAG, cytosine–adenine–guanine expansion; CAP_E_ = (age at entry) × (CAG − 33.66). Mean (SD) is presented for a continuous variable and frequency (percentage) for a categorical or count variable*.

### Statistical analysis

The main analysis focused on change over time in each premanifest group controlling for covariates (age, education, gender, depressed mood severity, brain scanner field strength). Interest was in the comparison of premanifest and control groups. Using linear mixed effects regression (LMER) (Verbeke and Molenberghs, 2000), 39 variables of interest were analyzed separately. Detailed descriptions of the variables are provided in the Supplementary Material. To control for site-to-site variability, a three-level model was used with repeated measures nested within participants, nested within sites. A preliminary analysis not presented showed evidence that linear curves were adequate for the modeling of change over time stratifying on CAP_E_ group. Random intercepts and slopes were specified for participants, as well as for sites. The time metric for the analysis was duration in the study (years in the study) with 0 = study entry. Two models were estimated for each outcome variable, a null model of duration and covariates only, and a full model adding CAP_E_ group intercept and slope differences. Maximum likelihood (ML) methods were used for estimation, which yield unbiased estimates under the widely-applicable assumption that the missing data mechanism is ignorable (Little and Rubin, 2002). The two models (null, full) were compared using the likelihood ratio test (LRT), which evaluates the null hypothesis that two nested models are statistically equivalent. The LRT statistic can be treated as an effect size measure in this case because the degrees of freedom are constant for each model test, and the outcome variables were rank-ordered according to the LRT statistic. To facilitate comparisons among the outcome variables, the estimated slopes from the LMER analysis were expressed in standard deviation (SD) units. To produce these estimates, each outcome was scaled using the grand mean and SD prior to the LMER analysis. Interest was in the comparison of premanifest and control groups. *Z*-tests of slope differences were computed as the estimated difference divided by its standard error. The control group *Z*-test was a test against a zero slope value. Three ancillary analyses were conducted. The first analysis examined possible effects of conversion. The second focused on required sample size for a hypothetical RCT. The third was a graphical analysis of trends over all progression periods for eight of the key variables. Variables were chosen conceptually to represent the phenotypic characteristics of HD (motor, cognitive, psychiatric) as well as to represent biological (imaging) and functional outcomes. Details of all analyses are presented in the Supplementary Material.

## Results

Table 2 shows the LMER results. The variables with the largest effect sizes (LRT statistics) were imaging measures based on regional brain volumes (corrected for intra-cranial volume and controlled for change in field strength). The two top-ranked measures were the putamen and caudate structure volumes. The slopes for the controls showed significant decrease over time (consistent with normal aging), but the decline in the gene-expansion groups steadily decreased over time for all groups. The slope for each premanifest group was statistically different from the Control group (all *p*s < 0.001). Other imaging variables demonstrating significant change relative to controls included accumbens, cerebral spinal fluid (CSF), lobar gray, hippocampus, and lobar white (though only for the High group). The putamen, caudate, CSF, and lobar gray measures showed significant longitudinal change in all three premanifest groups. A graphical depiction of change in brain volume for the groups is shown in Figure S1 in Supplementary Material.

**Table 2:** **Linear mixed effects regression results showing annual rate of change (slope) and rank-order based on effect size**.

Variable	Type	*N*	N*	Progression group				LRT	Rank
				Control	Low	Medium	High	
Putamen	Imaging	1206	2845	−0.0407***	−0.0900***	−0.1122***	−0.1119***	714.38***	1
Caudate	Imaging	1207	2846	−0.0331**	−0.0769***	−0.1028***	−0.1192***	515.33***	2
Accumbens	Imaging	1207	2846	−0.0264	−0.0279	−0.0853***	−0.1110***	417.38***	3
CSF	Imaging	1205	2840	0.0511***	0.0804**	0.1021***	0.0978***	223.19***	11
Lobar gray	Imaging	1175	2768	−0.0874***	−0.1220**	−0.1293***	−0.1523***	125.23***	19
Hippocampus	Imaging	1206	2845	−0.0337**	−0.0275	−0.0626**	−0.0910***	98.33***	23
Lobar white	Imaging	1149	2680	0.0223	0.021	0.0122	−0.0220***	63.73***	29
Thalamus	Imaging	1204	2843	−0.0327*	−0.0217	−0.0362	−0.0487	18.69**	39
TMS	Motor	1308	6077	0.0016	0.0332	0.0933***	0.2397***	415.75***	4
Chorea	Motor	1310	6124	0.0028	0.0395	0.0935***	0.2430***	332.72***	5
Brady	Motor	1308	6102	−0.003	0.0297	0.0790***	0.2063***	331.14***	6
Ocular	Motor	1309	6124	0.0008	0.0097	0.0683***	0.1397***	293.61***	8
Dystonia	Motor	1310	6130	−0.0009	0.0295	0.0524*	0.1698***	114.56***	21
Rigidity	Motor	1310	6127	0.0203	0.0406	0.028	0.0744***	38.58**	36
SDMT	Cognitive	1230	5252	0.0287**	−0.0012**	−0.0336***	−0.0800***	303.63***	7
Dysrhythmia	Cognitive	969	2270	−0.0107	−0.0089	0.0468*	0.1279***	236.60***	9
Stroop-Co	Cognitive	1228	5246	0.0327***	0.0103	−0.0284***	−0.0811***	234.50***	10
Stroop-Wo	Cognitive	1228	5255	0.0056	−0.0201*	−0.0416***	−0.0986***	222.97***	12
Sp-Tapping	Cognitive	975	2278	0.0046	0.038	0.0629**	0.1476***	214.97***	13
Smell-ID	Cognitive	1212	3745	0.0004	−0.0372*	−0.0559***	−0.1283***	189.31***	14
TMT-B	Cognitive	1221	3875	−0.02	0.0112	0.0188*	0.0878***	178.52***	15
Stroop-In	Cognitive	1228	5245	0.0452***	0.0252	0.0068**	−0.0468***	172.69***	16
EmoRec	Cognitive	978	2300	0.0342	0.049	0.0466	−0.0302**	163.05***	17
TMT-A	Cognitive	1223	3905	−0.0385*	0.0024*	0.0199**	0.1048***	141.71***	18
TFC	Functional	1308	6140	−0.0184	−0.0443	−0.0870***	−0.2093***	124.03***	20
ECog-C	Functional	795	1599	−0.0172	0.0469*	0.0576**	0.1074***	68.24***	26
ECog-P	Functional	899	1928	−0.0228	0.0292*	0.0117	0.0408**	50.05**	32
FAS	Functional	1052	4160	−0.0071	−0.0417	−0.0692*	−0.1688***	43.20***	35
WHODAS-C	Functional	708	1254	0.0105	0.0597	0.0859*	0.1193***	37.56**	37
WHODAS-P	Functional	769	1499	0.0199	0.0691	0.0522	0.0328	33.04**	38
S-OC-C	Psychiatric	1236	5392	−0.0293*	0.0027	0.0175**	0.0649***	100.54***	22
F-Exc-C	Psychiatric	1235	5288	−0.0226	−0.0036	0.0288**	0.0705***	97.95***	24
F-Apa-C	Psychiatric	1235	5288	−0.0158	0.0131	0.0346**	0.0706***	80.71***	25
S-GSI-C	Psychiatric	1237	5395	−0.0398***	−0.0096	−0.0016*	0.0404***	66.53***	27
S-Dep-C	Psychiatric	1236	5392	−0.0391**	−0.0054	−0.0048*	0.0352***	65.15***	28
S-Anx-C	Psychiatric	1237	5393	−0.0339**	−0.01	−0.0031*	0.0345***	53.14***	30
BDI	Psychiatric	1042	4120	−0.0063	−0.0044	−0.04	0.0191	52.88**	31
F-Dis-C	Psychiatric	1235	5288	−0.0219	0.0137*	0.0295***	0.0575***	49.57***	33
S-Hos-C	Psychiatric	1236	5392	−0.0256*	−0.0208	−0.0063	0.0166**	46.45**	34

Imaging variables adjusted for baseline intra-cranial volume. *N*, sample size; N*, number of data points; LRT, likelihood ratio test statistic; *p < 0.05, **p < 0.01, ***p < 0.001. Control p-value is a test against a zero slope and a CAP group p-value is a test against the Control slope. Progression Group (CAP group) is defined at study entry (CAP_e_). Progression Group columns show the standardized annual rate of change (slope). Rank (last column) is based on the likelihood ratio test statistic. CSF, cerebral spinal fluid; TMS, total motor score; Brady, bradykinesia; SDMT, Symbol Digit Modalities Test; Stroop-Co, Stroop Color and Word Test – color condition; Stroop-Wo, Stroop Color and Word Test – word condition; SP-Tapping, speeded tapping; Smell-ID, University of Pennsylvania Smell Identification Test (UPSIT); TMT-B, Trail Making Test, Part B; Stroop-In, Stroop Color and Word Test – interference condition; EmoRec, emotion recognition test; TMT-A, Trail Making Test, Part A; TFC, total functional capacity; ECog-C, Everyday Cognition Rating Scale – Companion Rating Scale; ECog-P, Everyday Cognition Rating Scale – Participant Rating Scale; FAS, functional activity scale; WHODAS-C, World Health Organization Disability Assessment Schedule – companion rating scale; WHODAS-P, World Health Organization Disability Assessment Schedule – participant rating scale; S-OC-C, Symptom Checklist 90–obsessive-compulsive scale – companion rating scale; F-Exc-C, Frontal Systems Behavioral Scale – executive subscale – companion rating scale; F-Apa-C, Frontal Systems Behavioral Scale – apathy subscale – companion rating scale; S-GSI-C, Symptom Checklist 90 – Global Severity Index – companion rating scale; S-Dep-C, Symptom Checklist 90 – depression subscale – companion rating scale; S-Anx-C, Symptom Checklist 90 – anxiety subscale – companion rating scale; BDI, Beck Depression Inventory–II; F-Dis-C, Frontal Systems Behavioral Rating Scale – disinhibition subscale – companion rating scale; S-Hos-C, Symptom Checklist 90 – hostility subscale – companion rating scale.

Total motor score (TMS) from the Unified Huntington’s Disease Rating Scale (UHDRS) showed the next highest effect size in change rates over time (rank four). The Control group slope was not statistically different from zero, and the Low group slope was not statistically different from the Control group slope. The Medium group slope was significantly larger than the Control slope, as was the High slope (all *p*s < 0.001). The next strongest effects were for bradykinesia (rank five) and chorea (rank six).

Decline in cognitive performance was significant in every measure examined. Symbol Digit Modality Test (SDMT) had the seventh-strongest effect over all measures. The Control group slope was positive and significantly greater than zero, indicating a practice effect. The Low group slope was negative and worse than the Control group slope. The Medium slope showed greater decline than the Low slope, and the High slope showed even greater decline. All cognitive measures examined showed significant changes in the High group, and 9 of 10 cognitive measures showed significant change in the Medium group. Four cognitive measures (SDMT, Stroop–word, Smell–ID, and the Trail Making Test) showed significant change over time in the Low group.

Regarding the functional variables, every measure examined showed significant change over time compared with the controls, except for the participant-rated World Health Organization Disability Assessment Schedule (WHODAS). The total functional capacity (TFC) scale (rank 20) showed the largest effect in the High group. The Control group slope and the Low group slope were not statistically different than zero. The Medium group slope showed significant decline, and the High group slope even more so. Though the Everyday Cognition Rating Scale (ECog) companion total had a weaker effect size (rank 26), the Low group was statistically different than the Control group, as were the higher progression groups. Four of the six functional measures showed robust change in the Medium group and three showed change in the Low group. The functional measures most appropriate for the different stages of premanifest HD varied. Whereas the TFC showed the greatest effect size in the High group, the companion WHODAS and ECog showed greater change in the Medium and Low groups.

Eight of the nine psychiatric variables showed significant change over time relative to controls. The SCL-90 obsessive–compulsive scale had the strongest effect (rank 22). There was an increase in obsessive–compulsive signs over time as the progression group increased (Control through High). Frontal Systems Behavioral Scale (FRSBE) executive and apathy subscales, ranked 24 and 25, respectively, also showed robust effect sizes. Seven of nine measures showed significant change in the Medium group and one psychiatric measure (FRSBE disinhibition) showed significant longitudinal change in the Low group.

Table 3 shows the six variables that had a statistically significant acceleration of the slope for the participants who converted. The fourth column (acceleration) shows the value added to a slope in Table 2 to indicate the additional deterioration associated with conversion. The variables are sorted by proportionate increase, with dystonia having the largest change under conversion (approximately 2.1 times faster decline). The acceleration factor is most applicable for the High group because the largest proportion of conversion occurred in this group (see Table 1). The acceleration factors ranged from a mild added slope acceleration of 0.06 SD per year for TMS, to a strong acceleration of 0.20 SD per year for dystonia.

**Table 3:** **Slope acceleration effect of motor diagnosis**.

Variable	Type	Chisq	Acceleration	Increase
Dystonia	Motor	91.77***	0.191182	2.125979
Stroop-Co	Cognitive	42.02***	−0.07891	1.973548
FAS	Functional	26.80***	−0.15594	1.923999
SDMT	Cognitive	29.57***	−0.06183	1.773312
TFC	Functional	29.49***	−0.08748	1.417992
TMS	Motor	22.20***	0.055938	1.233343

*p < 0.05, **p < 0.01, ***p < 0.001. Stroop-Co, Stroop Color and Word Test – color condition; FAS, functional activity scale; SDMT, Symbol Digit Modalities Test; TFC, total functional capacity; TMS, total motor score.

Table 4 shows the single-group estimated sample size as a function of dropout percentage, effect size, and estimated parameters for a hypothetical RCT of efficacy consistent with guidelines for Phase II trials (The Lancet Neurology, 2012). Listed in the effect size columns (20%, etc.) are the estimated required sample size. Results are listed for variables that required *N*/2 < 3000 for a 20% effect and 20% dropout. CSF had the smallest single-group sample size (e.g., *N*/2 = 27 for a 70% effect with no dropout), followed by putamen, caudate, TMS, speeded tapping, and additional variables. An approximate 70% difference in slopes was obtained in a recent clinical efficacy trial for HD (Huntington Study Group, 2006). Sample sizes do not take into account change associated with normal aging and thus may overestimate the effect sizes for differences between treatment versus placebo groups for any treatment that only addresses disease-related change.

**Table 4:** **Estimated required sample size (right side) for a two-arm phase II randomized clinical trial**.

Variable	Type	Dropout (%)	Estimated parameters					Effect size					
			*β_S_*	*g*_11_	*g*_12_	*g*_22_	$\sigma_{e}^{2}$	20%	30%	40%	50%	60%	70%
CSF	Imaging	0	4.094	486.882	5.189	3.183	53.737	332	147	83	53	37	27
CSF	Imaging	10	4.094	486.882	5.189	3.183	53.737	357	159	89	57	40	29
CSF	Imaging	20	4.094	486.882	5.189	3.183	53.737	386	172	97	62	43	32
Putamen	Imaging	0	−0.100	0.810	−0.004	0.003	0.037	391	174	98	63	43	32
Putamen	Imaging	10	−0.100	0.810	−0.004	0.003	0.037	421	187	105	67	47	34
Putamen	Imaging	20	−0.100	0.810	−0.004	0.003	0.037	455	202	114	73	51	37
Caudate	Imaging	0	−0.111	0.666	0.005	0.003	0.078	628	279	157	101	70	51
Caudate	Imaging	10	−0.111	0.666	0.005	0.003	0.078	676	301	169	108	75	55
Caudate	Imaging	20	−0.111	0.666	0.005	0.003	0.078	732	325	183	117	81	60
TMS	Motor	0	1.498	21.338	5.008	3.050	16.789	981	436	245	157	109	80
TMS	Motor	10	1.498	21.338	5.008	3.050	16.789	1051	467	263	168	117	86
TMS	Motor	20	1.498	21.338	5.008	3.050	16.789	1131	503	283	181	126	92
Sp-Tapping	Cognitive	0	7.345	3255.107	296.690	72.380	554.870	1230	546	307	197	137	100
Sp-Tapping	Cognitive	10	7.345	3255.107	296.690	72.380	554.870	1318	586	330	211	146	108
Sp-Tapping	Cognitive	20	7.345	3255.107	296.690	72.380	554.870	1421	632	355	227	158	116
Hippocampus	Imaging	0	−0.022	0.066	0.000	0.000	0.006	1322	588	330	212	147	108
Hippocampus	Imaging	10	−0.022	0.066	0.000	0.000	0.006	1422	632	356	228	158	116
Hippocampus	Imaging	20	−0.022	0.066	0.000	0.000	0.006	1540	684	385	246	171	126
Chorea	Motor	0	0.480	1.383	0.401	0.335	2.675	1373	610	343	220	153	112
Chorea	Motor	10	0.480	1.383	0.401	0.335	2.675	1472	654	368	236	164	120
Chorea	Motor	20	0.480	1.383	0.401	0.335	2.675	1586	705	397	254	176	130
Brady	Motor	0	0.520	4.748	0.811	0.438	3.305	1467	652	367	235	163	120
Brady	Motor	10	0.520	4.748	0.811	0.438	3.305	1574	699	393	252	175	128
Brady	Motor	20	0.520	4.748	0.811	0.438	3.305	1698	754	424	272	189	139
Dysrhythmia	Cognitive	0	3.002	592.124	53.942	12.919	135.291	1676	745	419	268	186	137
Dysrhythmia	Cognitive	10	3.002	592.124	53.942	12.919	135.291	1801	800	450	288	200	147
Dysrhythmia	Cognitive	20	3.002	592.124	53.942	12.919	135.291	1945	864	486	311	216	159
Thalamus	Imaging	0	−0.058	0.316	−0.006	0.003	0.069	2077	923	519	332	231	170
Thalamus	Imaging	10	−0.058	0.316	−0.006	0.003	0.069	2230	991	557	357	248	182
Thalamus	Imaging	20	−0.058	0.316	−0.006	0.003	0.069	2407	1070	602	385	267	197
TFC	Functional	0	−0.156	0.518	0.038	0.054	0.551	2540	1129	635	406	282	207
TFC	Functional	10	−0.156	0.518	0.038	0.054	0.551	2722	1210	680	435	302	222
TFC	Functional	20	−0.156	0.518	0.038	0.054	0.551	2932	1303	733	469	326	239
SDMT	Cognitive	0	−0.906	124.336	0.742	0.904	20.943	2547	1132	637	408	283	208
SDMT	Cognitive	10	−0.906	124.336	0.742	0.904	20.943	2740	1218	685	438	304	224
SDMT	Cognitive	20	−0.906	124.336	0.742	0.904	20.943	2964	1317	741	474	329	242

*Effect size is the percentage difference in rate of change (slope) of the treated and untreated groups (see Supplementary Material). CSF, cerebral spinal fluid; TMS, total motor score; SP-Tapping, speeded tapping; Brady, bradykinesia; TFC, total functional capacity; SDMT, Symbol Digit Modalities Test*.

**Figure 1:**
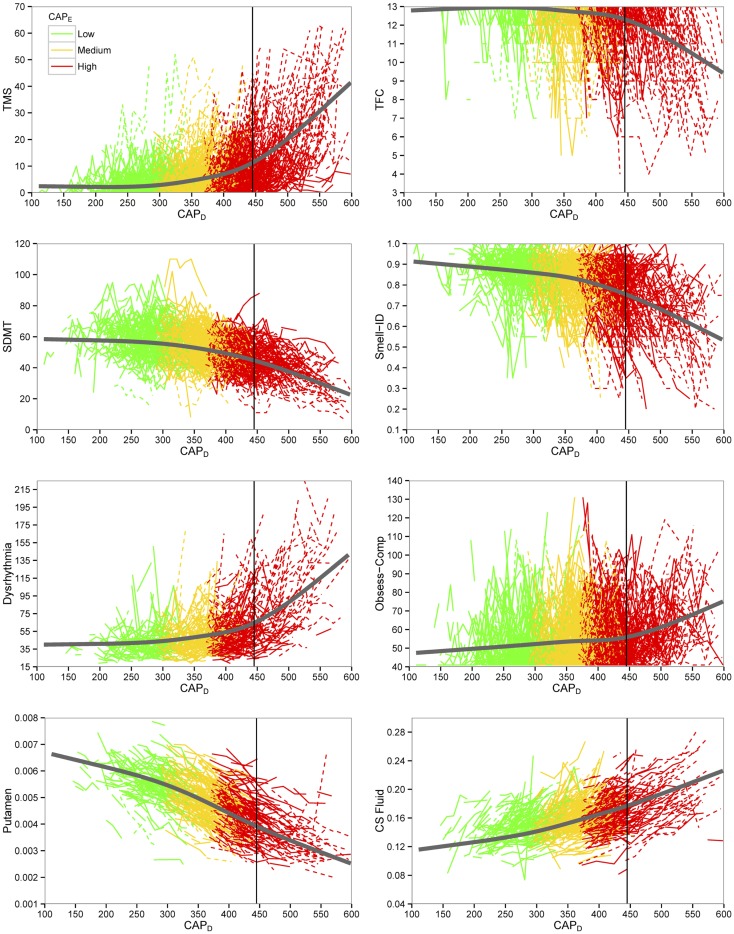
**Plots of key outcome variables for preventive clinical trials**. Empirical individual curves (thin lines) and fitted spline curves (thick lines) of key variables plotted over CAP_D_ (CAG-corrected age). Dashed lines indicate individuals who converted in the study. TMS indicates total motor score; TFC, total functional capacity; SDMT, Symbol Digit Modalities Test; Smell-ID, University of Pennsylvania Smell Identification Test (UPSIT); Obsess-Comp, obsessive-compulsive; CS Fluid, cerebral spinal fluid.

Figure 1 shows curves of individual participants (thin colored lines) and cubic spline curves (thick black lines) by premanifest stage. For some of the variables, change was slower for earlier premanifest stages but accelerated with proximity to average motor onset value (vertical line) with a greater acceleration thereafter.

**Figure 2:**
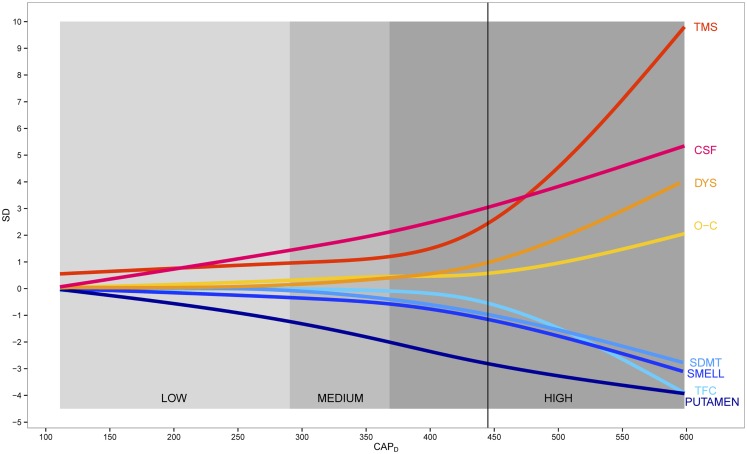
**Data-derived model of premanifest Huntington disease**. Scaled fitted spline curves of key variables plotted over CAP_D_ (CAG-corrected age). Blue colors indicate variables that decrease and red colors indicate variables that increase. Vertical axis is in standard deviation (SD) units. TMS indicates total motor score; CSF, cerebral spinal fluid; DYS, dysrhythmia; O–C, obsessive–compulsive; SDMT, Symbol Digit Modalities Test; SMELL, University of Pennsylvania Smell Identification Test (UPSIT); TFC, total functional capacity.

Figure 2 shows a model of disease progression for eight variables throughout the course of premanifest HD. The curves are based on a cubic spline fit after standardizing each variable relative to controls. The imaging, cognitive, and psychiatric variables showed linear increase over all premanifest stages, whereas motor and functional variables tended to show a non-linear trajectory with a sudden acceleration just prior to motor onset. Mean years in the study were 6, with a range of 1–10. Over 75% of the sample had >3 years of data, 15% had 2 years, and <10% had 1 year. A subset of *N* = 204 gene-expanded participants received a motor diagnosis during the study, referred to as “converters.” Dropout was less than 5% per year. Sample size variation was due to a number of historic study design events: 1) the NIH grant that funded the study was renewed three times and participants were recruited for the duration of each individual grant; and 2) grant reviewers increased sample size on each renewal, so the total sample size increased as the length of possible study duration decreased.

## Discussion

Findings show longitudinal change in 36 of 39 measures examined over a 10-year natural observation study in premanifest HD. Effect sizes suggest a preventive RCT could be efficiently designed to detect treatment effects in the neighborhood of 30%, and effects similar to the recent tetrabenazine efficacy trial (about 70% effect) might be found with sample sizes near to 30 per arm, depending on the amount of dropout (Huntington Study Group, 2006). Significant measures include each of the clinical phenotypic characteristics of HD (motor, cognitive, psychiatric), as well as biologic and functional outcomes. No previous study has so thoroughly documented a clinical battery for experimental therapeutics in stages of premanifest HD. Current findings dovetail with those generated from smaller studies. Most importantly, the specific measure chosen for each of the primary components typically measured in HD is dependent upon the disease stage of the premanifest cohort targeted for intervention. For example, the best cognitive variable for a clinical trial in persons who are <8 years to motor onset (High group) is tapping speed because it had the largest significant absolute value standardized slope (=0.1476). On the other hand, the most robust measure for tracking cognitive change in persons who are more than a dozen years to onset (Low group) is smell identification (see Table 2).

It is important to note that actual design of clinical trials involves many factors. Trial design will need to balance feasibility of cost-effective, multi-site research with the expense of advanced technology methods that may limit resources and result in fewer treatments being evaluated (Ross and Tabrizi, 2011). Ideally, clinical trials for HD will involve intervention at multiple and differing points along the cascade of changes that are known to occur from gene expansion to patient suffering. Hence, interference with the disease processes could involve gene silencing, altering posttranslational modifications of Huntington, amelioration of gene transcription abnormalities, or buttressing metabolic abnormalities to thwart the devastation that people with HD and their families endure. Different outcome measures may also have different kinds of utility for Phase I versus Phase II trials or symptomatic versus disease-modifying strategies. It is important to keep in mind that the measurements listed in Table 4 do not take into account changes related to normal aging. Changes in CSF volume, for example, are significant for premanifest participants but also demonstrate relatively large effect sizes for controls. Thus, a treatment that targets disease-related change, but not age-related change, may be better evaluated with measures that are more sensitive to longitudinal differences between premanifest cases versus controls.

Much recent attention has been devoted to the importance of natural history studies in the design of clinical trials^1^. In HD literature, several authors have developed conceptual models of natural history of the disease. None of the models are based on actual data, however (see text footnote 1). Findings from this study were used to develop a natural history model that is based on up to 10 years of natural observation spanning a substantial range of the premanifest period. The model can be used to develop progression-based care guidelines as well as to design clinical trials. Importantly, these data can provide external historical controls facilitating single-treatment-group or dose-selection trials without an active randomized placebo group (Elm et al., 2005; Czaplinski et al., 2006). Figure 2 provides a data-driven model of disease progression and illustrates progression using at least one variable from each clinical domain (motor, cognitive, psychiatric), biological evidence of decline (imaging variables), as well as a functional variable to encompass regulatory requirements. For practical purposes we chose outcomes that illustrated the greatest change for the High group, although the best choice for any specific clinical trial will vary dependent upon the treatment, the target and the premanifest cohort. For instance, the finding that the TFC did not show change in the Low and Medium groups, coupled with the sample sizes necessary to show a drug effect on TFC in the High group suggests that this measure is not likely to be an appropriate candidate for premanifest intervention in early-phase trials (for Phase 3, the Food and Drug Administration [FDA] wants to see a functional outcome). Efforts to provide a clinically meaningful outcome (as requested by regulatory agencies) may require consideration of other markers and extensive discussions with regulators. Such findings have immediate implications for current design of RCTs.

A primary strength of the PREDICT-HD study is that over 1300 gene-mutation-tested participants were prospectively followed up to, and, for some, through the point of actual motor diagnosis. Such data can be effective to document phenotypic and biologic changes that occur in persons with the gene expansion over the decade prior to, and, just after the manifestation of disease. Findings suggest the course of biologic progression in premanifest HD appears linear for imaging, cognitive, and psychiatric data, but non-linear for motor and functional data (see Figure 2). Motor expression appears to accelerate as the disease manifests over the course of approximately 15 years prior to motor onset. It is important to note that increases in the number and severity of clinical outcomes reflect measurement aspects of the manifestation of disease and do not necessarily reflect a curvilinear, or increased, rate of disease progression. One possible explanation may be that atrophy of each individual brain region (e.g., putamen as shown in Figures 1 and 2) proceeds relatively linearly, but as additional brain regions begin to undergo degeneration and dysfunction, their combined effect causes acceleration of the clinical expression of disease.

Additional strengths include the large, worldwide collaboration among 32 sites and across brain scanners, cultures, and languages, involving multiple disciplines and specialties. The PREDICT-HD study may be most relevant to actual clinical trials where multiple sites are likely to be used to acquire sufficient sample sizes (The Lancet Neurology, 2012). Finally, the care with which the study was conducted provides quality control, quality assurances, standardization protocols, and statistical control for many common confounds (age, gender, education) and not-so-common confounds (depressed mood, field strength). All components of the study are shared worldwide to assure findings can be replicated and utilized in the future as more knowledge is acquired about HD and other neurodegenerative disorders, so PREDICT-HD data can continue to facilitate progress for decades onward.

Weaknesses also exist in the study. Interval to follow-up was 1 year for clinical and cognitive assessment and 2 years for imaging, and clinical trials demand more frequent assessments. It is recommended that the measures proposed be subjected to a brief repeated measures study over a period of 6 months to assure that more rapid assessment can be documented. Finally, due to the length of the PREDICT-HD study, some protocol changes were unavoidable. A common-but-important variation in our study was the change in MRI scanners from 1.5 to 3 T. The basal ganglia structures were processed to accommodate this change, and field strength was statistically controlled in the analysis. Solutions to varying field strength and other methodological challenges of PREDICT-HD can be made available to researchers who are interested in natural history studies. The lobar white matter measures reported in this work incorporate data from much more heterogeneous data collection than were reported in previous publications. The expanded subject inclusion for lobar white matter measures included both the 1.5 and 3 T scanners from a larger number of scanning sites. While the strength of white matter lobar measures is less than previously reported, we believe this is due to inherent measurement variability introduced by heterogeneous data collection. Systematically addressing the variability due to heterogeneous data collection is a focus of ongoing work.

## Author Contributions

Study concept and design, Jane S. Paulsen, Jeffrey D. Long, Hans J. Johnson, Elizabeth H. Aylward, Christopher A. Ross; acquisition of data, Jane S. Paulsen, Hans J. Johnson, Elizabeth H. Aylward, Christopher A. Ross, Janet K. Williams, Martha A. Nance, Cheryl J. Erwin, Holly J. Westervelt, H. Jeremy Bockholt, Elizabeth A. McCusker, Edmond M. Chiu, Peter K. Panegyres, PREDICT-HD Investigators and Coordinators of the Huntington Study Group; analysis and interpretation of data, Jane S. Paulsen, Jeffrey D. Long, Elizabeth H. Aylward, Christopher A. Ross, Martha A. Nance, Cheryl J. Erwin, Deborah L. Harrington, H. Jeremy Bockholt, Ying Zhang; drafting of the manuscript, Jane S. Paulsen, Jeffrey D. Long; critical revision of the manuscript for important intellectual content, Jane S. Paulsen, Jeffrey D. Long, Hans J. Johnson, Elizabeth H. Aylward, Christopher A. Ross, Janet K. Williams, Martha A. Nance, Holly J. Westervelt, Deborah L. Harrington, H. Jeremy Bockholt, Elizabeth A. McCusker, Peter K. Panegyres; statistical analysis, Jane S. Paulsen, Jeffrey D. Long; obtained funding, Jane S. Paulsen; study supervision, Jane S. Paulsen, Hans J. Johnson, H. Jeremy Bockholt.

## Conflict of Interest Statement

Jane S. Paulsen, Hans J. Johnson, Martha A. Nance, Elizabeth H. Aylward, Christopher A. Ross, and Janet K. Williams received grants from the National Institutes of Health. Jane S. Paulsen, Martha A. Nance, and Elizabeth H. Aylward received grants from CHDI Foundation, Inc. Jeffrey D. Long, Cheryl J. Erwin, Holly J. Westervelt, Deborah L. Harrington, Ying Zhang, Elizabeth A. McCusker, Edmond M. Chiu, Peter K. Panegyres, and H. Jeremy Bockholt have reported no commercial or financial relationships that could be construed as potential conflicts of interest.

## Supplementary Material

The Supplementary Material for this article can be found online at http://www.frontiersin.org/Journal/10.3389/fnagi.2014.00078/abstract

## Footnotes

^1^Workshop on Natural History Studies of Rare Diseases: meeting the Needs of Drug Development and Research.” [U.S. Department of Health & Human Services, National Institutes of Health and U.S. Food and Drug Administration workshop, Bethesda, MD, USA May 16–17, 2012, https://events-support.com/events/Natural_History_Studies/page/87]

## Notes

**PREDICT-HD Investigators and Coordinators of the Huntington Study Group:** *Investigators, Coordinators, Motor Raters, and Cognitive Raters:* Stephen Cross, Patricia Ryan, and Eric A. Epping (University of Iowa, Iowa City, IA, USA); Edmond M. Chiu, Joy Preston, Anita Goh, Stephanie Antonopoulos, and Samantha Loi (St. Vincent’s Hospital, The University of Melbourne, Kew, VIC, Australia); Lynn Raymond, Joji Decolongon, Mannie Fan, and Allison Coleman (University of British Columbia, Vancouver, BC, Canada); William M. Mallone and Greg Suter (Hereditary Neurological Disease Centre, Wichita, KS, USA); Christopher A. Ross, Mark Varvaris, and Nadine Yoritomo (John Hopkins University, Baltimore, MD, USA); Elizabeth A. McCusker, Jane Griffith, Clement Loy, and David Gunn (Westmead Hospital, Sydney, NSW, Australia); Mark Guttman, Alanna Sheinberg, and Albie Law (Centre for Addiction and Mental Health, University of Toronto, Markham, ON, Canada); Kimberly Quaid, Melissa Wesson, and Joanne Wojcieszek (Indiana University School of Medicine, Indianapolis, IN, USA); Joel Perlmutter, Stacey Barton, and Shineeka Smith (Washington University, St. Louis, MO, USA); Roger A. Barker, Sarah Mason, and Natalie Valle Guzman (Cambridge Centre for Brain Repair, Cambridge, UK); Susan Perlman and Brian Clemente (UCLA Medical Center, Los Angeles, CA, USA); Randi Jones, Cathy Wood-Siverio, and Stewart A. Factor (Emory University School of Medicine, Atlanta, GA, USA); Ali Samii and Alma Macaraeg (University of Washington and VA Puget Sound Health Care System, Seattle, DC, USA); Peter K. Panegyres, Joseph Lee, Maria Tedesco, and Brenton Maxwell (Neurosciences Unit, Graylands, Selby-Lemnos & Special Care Health Services, Perth, WA, Australia); Rajeev Kumar, Diane Erickson, and Breanna Nickels (Colorado Neurological Institute, Englewood, CO, USA); Frederick Marshall, Amy Chesire, Mary Wodarski, and Charlyne Hickey (University of Rochester, Rochester, NY, USA); Michael D. Geschwind, Sharon Sha, and Gabriela Satris (University of California San Francisco, CA, USA); Anwar Ahmed, Christine Reece, Alex Bura, Lyla Mourany, and Jagan Pillai (Cleveland Clinic Foundation, Cleveland, OH, USA); Pietro Mazzoni, Karen Marder, and Paula Wasserman (Columbia University Medical Center, New York, NY, USA); David Craufurd, Judith Bek, and Elizabeth Howard (University of Manchester, Manchester, UK); Tom Warner and Maggie Burrows (National Hospital for Neurology and Neurosurgery, London, UK); Michael Orth, Sigurd Süβmuth, Katrin Barth, Sonja Trautmann, Daniela Schwenk, and Carolin Eschenbach (University of Ulm, Ulm, Germany); Vicki Wheelock, Lisa Kjer, Amanda Martin, and Sarah Farias (University of California, Davis, Sacramento, CA, USA); Zosia Miedzybrodzka, Daniela Rae, and Mariella D’Alessandro (Clinical Genetics Centre, Aberdeen, Scotland, UK); Oksana Suchowersky, Phyllis Chua, and Angela Komiti (The University of Melbourne, Royal Melbourne Hospital, Melbourne, VIC, Australia); Diana Rosas (Massachusetts General Hospital, Boston, MA, USA); Anne Rosser, Kathy Price, and Sarah Hunt (Cardiff University, Cardiff, Wales, UK); Joseph Jankovic and William Ondo (Baylor College of Medicine, Houston, TX, USA); Wayne Martin, Pamela King, Marguerite Wieler, and Satwinder Sran (University of Alberta, Edmonton, AB, Canada); Martha A. Nance (University of Minnesota/Minnesota VA Medical Center, Minneapolis, MN, USA); Justo Garcia de Yébenes (Hospital Ramón y Cajal, Madrid, Spain); Richard Dubinsky (University of Kansas Medical Center, Kansas City, KS, USA).

*Executive Committee*: Jane S. Paulsen, Principal Investigator, Eric A. Epping, Jeffrey D. Long, Hans J. Johnson, H. Jeremy Bockholt, and Kelsey Montross.

*Scientific Consultants: Brain*: Jean Paul Vonsattel and Carol Moskowitz (Columbia University Medical Center); *Cognitive:* Deborah L. Harrington (University of California, San Diego, San Diego, CA, USA); Tamara Hershey (Washington University); and Holly J. Westervelt (Rhode Island Hospital/Alpert Medical School of Brown University, Providence, RI, USA); *Functional:* Janet K. Williams and Nancy Downing (University of Iowa); *Imaging:* Hans J. Johnson (University of Iowa); Elizabeth H. Aylward (Seattle Children’s Research Institute, Seattle, DC, USA); Christopher A. Ross (Johns Hopkins University); and Vincent A. Magnotta (University of Iowa); *Psychiatric:* Eric A. Epping (University of Iowa); David Craufurd (University of Manchester);

*Core sections: Biostatistics:* Jeffrey D. Long, Ji-In Kim, James A. Mills, Ying Zhang, Dawei Liu, Wenjing Lu, and Spencer Lourens (University of Iowa); *Ethics:* Cheryl J. Erwin (McGovern Center for Health, Humanities and the Human Spirit); Eric A. Epping and Janet K. Williams (University of Iowa); Martha A. Nance (University of Minnesota); *Biomedical informatics:* H. Jeremy Bockholt and Ryan Wyse (University of Iowa).
