# Supplementary material for: Clinical and Biomarker Changes in Premanifest Huntington Disease Show Trial Feasibility: A Decade of the PREDICT-HD Study
Source: Front Aging Neurosci. 2014 Apr 22;6:78. doi: 10.3389/fnagi.2014.00078 (PMC4000999; doi:10.3389/fnagi.2014.00078)
Supplement: Supplementary file 1 [file Data_Sheet1.PDF]

Supplementary Material

Clinical and biomarker changes in premanifest Huntington disease show trial feasibility: a decade of the PREDICT-HD study

Jane S. Paulsen[1, 2, 3*], Jeffrey D. Long[1, 4], Hans J. Johnson[1, 5], Elizabeth H. Aylward[6], Christopher A. Ross[7], Janet K. Williams[8], Martha A. Nance[9], Cheryl J. Erwin[10], Holly J. Westervelt [11], Deborah L. Harrington[12, 13], H. Jeremy Bockholt[14], Ying Zhang[15], Elizabeth A. McCusker[16], Edmond M. Chiu[17], Peter K. Panegyres[18], and the PREDICT-HD Investigators and Coordinators of the Huntington Study Group

[1]Department of Psychiatry, Carver College of Medicine, The University of Iowa, Iowa City, IA, USA
[2]Department of Neurology, Carver College of Medicine, The University of Iowa, Iowa City, IA, USA
[3]Department of Psychology, The University of Iowa, Iowa City, IA, USA
[4]Department of Biostatistics, College of Public Health, The University of Iowa, Iowa City, IA, USA
[5]Departments of Electrical and Computer Engineering and Biomedical Engineering, College of Engineering, The University of Iowa, Iowa City, IA, USA
[6]Center for Integrative Brain Research, Seattle Children's Research Institute, Seattle, WA, USA
[7]Division of Neurobiology, Johns Hopkins University, Baltimore, MD, USA
[8]College of Nursing, The University of Iowa, Iowa City, IA, USA
[9]Department of Neurology, College of Medicine, The University of Minnesota, Minneapolis, MN, USA
[10]McGovern Center for Humanities and Ethics, The University of Texas Medical School at Houston, Houston, TX, USA
[11]Department of Psychiatry & Human Behavior, Division of Biology and Medicine, Brown University, Providence, RI, USA
[12]Department of Radiology, University of California, San Diego, School of Medicine, San Diego, CA, USA
[13]Veterans Affairs San Diego Healthcare System, San Diego, CA, USA
[14]Advanced Biomedical Informatics Group, LLC, Iowa City, Iowa, USA
[15]Department of Biostatistics, Indiana University School of Medicine, Indianapolis, IN, USA
[16]Department of Neurology, Westmead Hospital, The University of Sydney, Sydney, New South Wales, Australia
[17]Department of Psychiatry, The University of Melbourne, Melbourne, Victoria, Australia
[18]Neurodegenerative Disorders Research Pty Ltd, Perth, Western Australia, Australia

***Correspondence:** Jane S. Paulsen, Ph.D., Carver College of Medicine, Carver Chair in Neuroscience and Professor of Neurology, Psychiatry, and Psychology, The University of Iowa, 1-305 Medical Education Building, Iowa City, IA 52242-1000, USA.
predict-publications@uiowa.edu; jane-paulsen@uiowa.edu

1. Variable Descriptions

Lobar white = the sum of frontal, parietal, temporal, and occipital lobe white matter for both hemispheres divided by baseline intra-cranial volume (ICV).

Lobar gray = the sum of frontal, parietal, temporal, and occipital lobe gray matter for both hemispheres divided by baseline ICV.

CSF = cerebral spinal fluid divided by baseline ICV.

Hippo = hippocampus divided by baseline ICV.

TMS = total motor score from the Unified Huntington Disease Rating Scale (UHDRS). Standardized ratings of oculomotor function, dysarthria, chorea, dystonia, gait and postural stability (Huntington Study Group, 1996).

Brady = bradykinesia subscale from the UHDRS. Rating of abnormal slowness or rigidity of movement (Huntington Study Group, 1996).

Ocular = ocular subscale from the UHDRS. Rating of eye movement and tracking (Huntington Study Group, 1996).

SDMT = Symbol Digit Modalities Test. The SDMT is an adaptation of the Wechsler Digit Symbol subtest that measures working memory, complex scanning, and processing speed (Wechsler, 1981; Lezak et al., 2004). Participants use a key presented at the top of the test page to match symbols with numbers presented in horizontal rows. The task requires that the participant fill in the appropriate symbols below the matching numbers as quickly as possible. Raw scores indicate the number of items correctly completed in 90 seconds (Smith, 1982).

Stroop-Co = Stroop Color and Word Test – color condition. The Stroop Color and Word Test consists of three 45-second trials (Stroop, 1935). The first two trials (color identification and word reading) measure basic attention. In the first trial, participants must correctly identify the color of ink patches on a stimulus card. In the second trial, participants read the names of colors printed in black ink. In the third trial, the interference trial, participants must consistently inhibit an overlearned response by identifying the color of ink (red, green, blue) that the stimulus color words are printed in rather than reading the word aloud. Raw scores indicate the number of items correctly completed per trial (Golden, 1978).

Timing = time production or paced tapping. Participants were presented with a 1.8 Hz tone and were instructed to tap along with it when ready. After 11 more presentations of the tone, the tone stopped, and participants attempted to continue to tap at the same pace for 31 more taps. The variable analyzed is the reciprocal of the standard deviation of the intertap interval for an alternating thumbs trial (smaller values indicate worse performance) over five trials (Rowe et al., 2010).

Stroop-Wo = Stroop Color and Word Test – word condition. The Stroop Color and Word Test consists of three 45-second trials (Stroop, 1935). The first two trials (color identification and word reading) measure basic attention. In the first trial, participants must correctly identify the color of ink patches on a stimulus card. In the second trial, participants read the names of colors printed in black ink. In the third trial, the interference trial, participants must consistently inhibit an overlearned response by identifying the color of ink (red, green, blue) that the stimulus color words are printed in rather than reading the word aloud. Raw scores indicate the number of items correctly completed per trial (Golden, 1978).

Sp-Tapping = speeded tapping. Finger tapping speed was assessed by calculating the mean intertap interval of five 10-second trials of tapping as quickly as possible with the nondominant finger (smaller values indicate better performance) (Rowe et al., 2010).

Smell-ID = University of Pennsylvania Smell Identification Test (UPSIT). The smell identification test is a multiple-choice measure of olfactory recognition. Participants scratched a scented patch in a test booklet and identified the corresponding scent label from four multiple choice options (Doty et al., 1984). Some participants completed the full four-booklet version of the UPSIT, others completed an abbreviated, 20-item version. The percentage of correctly identified scents was analyzed (Doty et al., 1984).

TMT-B = Trail Making Test, Part B. In TMT-B, participants alternate between connecting numbered and lettered circles according to ascending, alphabetical order (i.e., 1-A, 2-B, 3-C, etc.). Raw scores indicate the number of seconds required to complete each test (Reitan, 1958; O'Rourke et al., 2011).

Stroop-In = Stroop Color and Word Test – interference condition. The Stroop Color and Word Test consists of three 45-second trials (Stroop, 1935). The first two trials (color identification and word reading) measure basic attention. In the first trial, participants must correctly identify the color of ink patches on a stimulus card. In the second trial, participants read the names of colors printed in black ink. In the third trial, the interference trial, participants must consistently inhibit an overlearned response by identifying the color of ink (red, green, blue) that the stimulus color words are printed in rather than reading the word aloud. Raw scores indicate the number of items correctly completed per trial (Golden, 1978).

EmoRec = emotion recognition test. Emotion recognition was assessed by two emotion-labeling tasks (Johnson et al., 2007). One of the tasks employed static photographs of human faces, while the other used the same stimuli with simulated movement (Ekman and Friesen, 1976). In both tasks, participants were asked to identify the emotion displayed by a target face. In the static condition, an expression of moderate intensity was presented for one second. The options were fear, disgust, happy, sad, surprise, anger, and neutral. In the simulated movement condition, an expression of mild intensity presented for 500 milliseconds transformed into an expression of moderate intensity for 500 milliseconds. The variables analyzed for each are the number of negative emotions correctly identified (Johnson et al., 2007; Aylward et al., 2011).

TMT-A = Trail Making Test, Part A. In TMT-A, participants draw lines connecting numbered circles as quickly as possible. Raw scores indicate the number of seconds required to complete each test (Reitan, 1958; O'Rourke et al., 2011).

TFC = total functional capacity from the UHDRS. A list of independent and common daily tasks that can be accomplished (Huntington Study Group, 1996).[1]

ECog-C = Everyday Cognition Rating Scale – Companion Rating Scale. An adult familiar with the participant rates the participant's memory, language, semantic knowledge, visuospatial abilities, planning, organization, and divided attention (Farias et al., 2008).

FAS = functional activity scale from the UHDRS (Huntington Study Group, 1996).

WHODAS-C = World Health Organization Disability Assessment Schedule – companion rating scale. A generic assessment for health and disability as related by a close companion (World Health Organization, 1988).

S-OC-C = Symptom Checklist 90 – obsessive compulsive scale – companion rating scale. A 90-item assessment taking 15 minutes to administer, with this subscale focusing on obsessive-compulsive disorders as rated by companions (Derogatis, 1977).

F-Exc-C = Frontal Systems Behavioral Scale – executive subscale – companion rating scale. Part of a 46-item behavior rating scale focusing on abstraction, problem solving, and hypothesis generation as rated by a companion focusing on dorsolateral prefrontal circuitry (Grace and Malloy, 2000).

F-Apa-C = Frontal Systems Behavioral Scale – apathy subscale – companion rating scale. Part of a 46-item behavior rating scale associated with anterior cingulate circuitry (Grace and Malloy, 2000).

S-GSI-C = Symptom Checklist 90 – Global Severity Index – companion rating scale. A 90-item assessment taking 15 minutes to administer. Global severity is one of the three major indices (Derogatis, 1977).

S-Dep-C = Symptom Checklist 90 – depression subscale – companion rating scale. A 90-item assessment taking 15 minutes to administer, with this subscale focusing on depressive symptoms as rated by companions (Derogatis, 1977).

S-Anx-C = Symptom Checklist 90 – anxiety subscale – companion rating scale. A 90-item assessment taking 15 minutes to administer, with this subscale focusing on anxiety as rated by companions (Derogatis, 1977).

BDI = Beck Depression Inventory–II. A 21-question inventory to measure the severity of depression (Beck et al., 1993).

F-Dis-C = Frontal Systems Behavioral Rating Scale – disinhibition subscale – companion rating scale. Part of a 46-item scale associated with orbitofrontal circuitry (Grace and Malloy, 2000).

S-Hos-C = Symptom Checklist 90 – hostility subscale – companion rating scale. A 90-item assessment taking 15 minutes to administer with this subscale focusing on outward hostility toward others as rated by companions (Derogatis, 1977).

2. Image Processing

Basal ganglia structures had different imaging processing than lobar white and lobar gray. We begin with a description of the former.

2.1 Basal Ganglia Structures

All imaging analysis was performed at the University of Iowa Scalable Informatics, Neuroimaging, Analysis, Processing, and Software Engineering (SINAPSE) laboratory. Acquired scans are processed through a fully automated procedure, Brain Research: Analysis of Images, Networks, and Systems (BRAINS) AutoWorkup (BAW) (Pierson et al., 2011), improved with SyN (Avants et al., 2008) registration from the Advanced Normalization Toolkit in the BRAINSTools software package.[1] All scans begin with visual inspection of the raw data, so only images of sufficient quality are processed. Each dataset, T1 and/or T2-weighted images, was processed together to improve the robustness of the procedure from complimentary information provided by multiple modalities. The best-rated T1-weighted image is spatially normalized to an "AC-PC" alignment, where the anterior commissure is located at physical location (0,0,0) based on prominent landmarks in MRI, including anterior and posterior commissure, and mid-sagittal plane (Ghayoor et al., 2013). The remaining scans acquired in the same session are then rigidly aligned to the spatially normalized T1 image, and simultaneously processed by the automated bias-field correction (ABC) algorithm, BRAINSABC[2]. For each given modality, BRAINSABC produces an average of independently bias-field corrected MR images resampled in 1mm x 1mm x 1mm, and their respective corresponding 17 tissue probability maps, including white matter, grey matter, and CSF. At this point, all longitudinal scan sessions for a single subject are used jointly to build a subject-specific atlas. The subject-specific atlas best represents the average longitudinal shape for that subject with respect to minimum mean square error of spatial displacement to each time point. This joint session template building step is used to maximize consistency by regularizing inherent scanner variation in longitudinal studies. The resulting data set of bias-corrected average T1 and/or T2 images are subsequently segmented for subcortical structures using an automated segmentation framework, BRAINSCut. BRAINSCut is an extension of previous work that now employs robust random forest machine learning that has been validated on multi-site MR data[3] (Powell et al., 2008). The subcortical structures of interest include nucleus accumbens, caudate, putamen, hippocampus, and thalamus. The results of this procedure were again visually inspected and resulted in a success rate greater than 90%. All the development processing was blinded to clinical data, such as HD gene-expansion status, gender, and age. Premanifest disease progression based on MRI is shown in **Supplementary Figure 1**. Four brain structures (putamen, caudate, hippocampus, thalamus) paneled by group and three biannual measurements are color coded to represent the group aggregate structural volume proportional to that of the controls at study entry. The Control and Low groups had volumes that were nearly indistinguishable at baseline, whereas the Medium and High groups had substantial loss of putamen and caudate volumes. Over time, the putamen and caudate become markedly lighter in color, especially for the Medium and High groups, showing volume loss that substantially outpaced the natural aging depicted in the Control panels. At Year 1, putamen volumes in the Low, Medium, and High group were at 98%, 86%, and 73%, respectively, of the Control volume. By Year 5 in the study, Low, Medium, and High putamen volumes were 93%, 79%, and 66%, respectively.

---

[1]"BRAINSTool Package". (https://github.com/BRAINSia/BRAINSTools).
[2]Kim, E. Y., and Johnson, H. J. (2013). Robust Multi-site MR Data Processing: Iterative Optimization of Bias Correction, Tissue Classification, and Registration. Submitted for Review.
[3]Kim, E. Y., Magnotta, V. A., Johnson, H.J., et al. (2013). Automated Segmentation Framework: Determining Robust Machine-Learning Algorithm and Intensity Normalization for Scalable Brain MRI. Submitted for review.

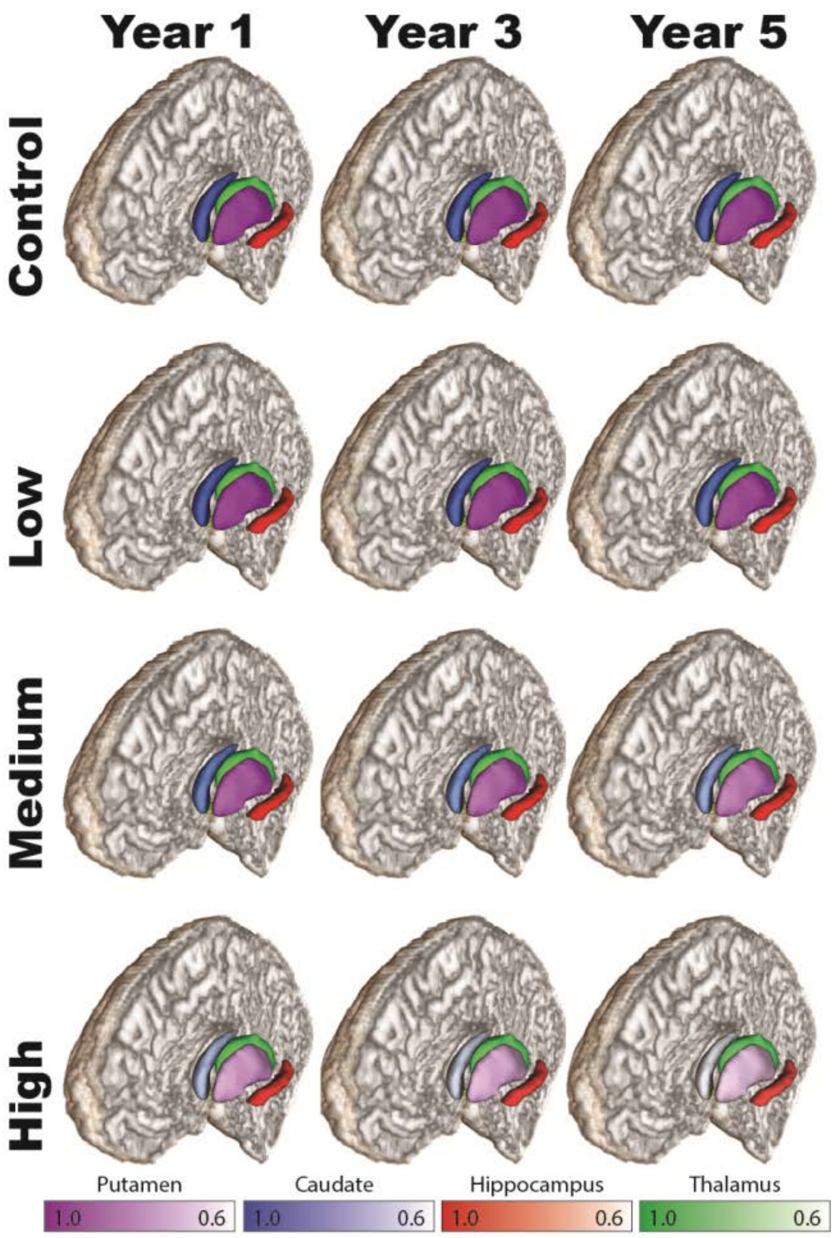

Supplementary Figure 1. Volume loss in premanifest HD. Brain structures by CAP_E group and year. Color gradient indicates volume relative to the Control group at Year 1 (lighter indicates volume loss relative to controls).

2.2 Lobar White and Lobar Gray

To extract reliable volumetric segmentations, images were automatically processed with the longitudinal stream (Reuter et al., 2012) in FreeSurfer which is documented and freely available for download (http://surfer.nmr.mgh.harvard.edu/). Specifically, an unbiased within-subject template space and image is created using robust, inverse consistent registration (Reuter et al., 2010). Several processing steps, such as skull stripping, Talairach transforms, atlas registration as well as spherical surface maps and parcellations are then initialized with common

information from the within-subject template, significantly increasing reliability and statistical power (Reuter et al., 2012). The technical details of these procedures are described in prior publications (Dale and Sereno, 1993; Dale et al., 1999; Fischl et al., 1999a; Fischl et al., 1999b; Fischl and Dale, 2000; Fischl et al., 2001; Fischl et al., 2002; Fischl et al., 2004a; Fischl et al., 2004b; Segonne et al., 2004; Han et al., 2006; Jovicich et al., 2006; Reuter et al., 2010; Reuter et al., 2012). Briefly, this processing includes removal of non-brain tissue using a hybrid watershed/surface deformation procedure (Segonne et al., 2004), automated Talairach transformation, segmentation of the subcortical white matter and deep gray matter volumetric structures (Fischl et al., 2002; Fischl et al., 2004a), intensity normalization (Sled et al., 1998), tessellation of the gray matter white matter boundary, automated topology correction (Fischl et al., 2001; Segonne et al., 2007), and surface deformation following intensity gradients to optimally place the gray/white and gray/cerebral spinal fluid borders at the location where the greatest shift in intensity defines the transition to the other tissue class (Dale and Sereno, 1993; Dale et al., 1999; Fischl and Dale, 2000). FreeSurfer morphometric procedures have been demonstrated to show good test-retest reliability across scanner manufacturers and across field strengths (Han et al., 2006; Reuter et al., 2012).

3. Statistical Analyses

3.1 Linear Mixed Effects Regression

The main analysis used linear mixed effects regression (LMER) (Verbeke and Molenberghs, 2000). Suppose that y_{ijk} is the response score for the kth time (k = 1, …, n_{ij}) for the jth participant (j = 1, …, N_i) and the ith site (i = 1, …, 32). The LMER model for a single outcome variable is

$$y_{ijk} = (\alpha + a_i + a_{ij}) + (\beta + b_i + b_{ij})t_{ijk} + \gamma_1 sex_{ij} + \gamma_2 educ_{ij} + \gamma_3 age_{ij1}$$
$$+\delta_1 d_{Lij} + \delta_2 d_{Mij} + \delta_3 d_{Hij} + \delta_4 d_{Lij} t_{ijk} + \delta_5 d_{M\,ij} t_{ijk} + \delta_6 d_{H\,ij} t_{ijk} + \epsilon_{ijk} \quad \text{(A1)}$$

where t is time in years since entry into the study, *sex* is a gender dummy code (1 if female, 0 if male), *educ* is years of education, *age* is age at study entry, d_L is a dummy code for the Low group (1 if in the group, 0 otherwise), d_M is a dummy code for the Medium group, and d_H is a dummy code for the High group. In Equation (A1), α is the fixed control group intercept; β is the fixed control group slope; γ_1, γ_2, γ_3, are the covariate fixed effects; δ_1, δ_2, δ_3 are the fixed CAP group intercept differences vs. controls; δ_4, δ_5, δ_6 are the fixed CAP group slope differences vs. controls; a_i and b_i are site random effects; a_{ij} and b_{ij} are participant random effects; and ϵ_{ijk} is random error. We assume $\begin{bmatrix} a_i \\ b_i \end{bmatrix} \sim \mathcal{N}(\mathbf{0}, \boldsymbol{G}) \perp \begin{bmatrix} a_{ij} \\ b_{ij} \end{bmatrix} \sim \mathcal{N}(\mathbf{0}, \boldsymbol{H}) \perp \epsilon_{ijk} \sim \mathcal{N}(0, \sigma_\epsilon^2 \boldsymbol{I}_{ij})$. An estimated CAP group slope in **Table 2** in the **main text** was computed as $\hat{\beta} + \hat{\delta}_l, l$ =4, 5, 6.

Because depression can affect cognitive performance, depression was added as an additional dynamic covariate when the outcome was a cognitive variable. Suppose dep is the dynamic depression score. Then the terms $\eta_1 dep_{ijk}$ and $\eta_2 dep_{ijk} t_{ijk}$ were added to Equation (A1), representing the intercept and slope effect of depression, respectively. For the imaging data, MRI

field strength varied over the duration of the study, with a change from 1.5T to 3T for some participants. For the imaging variables, field strength was modeled as a dynamic binary variable, d_{3T}, with $d_{3T} = 1$ if 3T and 0 if 1.5T. Similar to depression, the terms $\eta_1 d_{3T,ijk}$ and $\eta_2 d_{3T,ijk} t_{ijk}$ were added to Equation (A1), representing the intercept and slope effect of field strength, respectively. We set $d_{3T} = 0$ and $dep = 0$ when computing the slopes of **Table 2** in the **main text**, so that the slopes are for 1.5T with an imaging variable, and for completely non-depressed people with a cognitive variable.

An omnibus likelihood ratio test (LRT) of the null hypothesis $H_0: \delta_1 = \cdots = \delta_6 = 0$ was performed for each variable. The size of the LRT test statistic was used to rank the variables, as seen in **Table 2** in the **main text**. The CAP group *p*-values of **Table 2** were based on the *z*-test of the difference between a CAP group slope and the control slope, $Z = \hat{\delta}_l / SE(\hat{\delta}_l)$. The Control group *p*-value was based on the *z*-test against a zero slope, $Z = \hat{\beta} / SE(\hat{\beta})$.

3.2 Dynamic Diagnosis Model

The impact of motor diagnosis was modeled using a time-varying (dynamic) dummy code in LMER (Long, 2012). Because motor diagnosis can only occur for gene-expanded individuals, the controls were omitted from the analysis. Let $m = 1$ if motor diagnosis occurs and 0 otherwise at the kth time point for the jth subject at the ith site. Similar to the examples above, m was included in Equation (A1) as a main effect (single variable) and its product with time. This allowed separate intercepts and slopes for the repeated measures after motor diagnosis and prior to diagnosis.

3.3 Cubic Spline Model

LMER with natural cubic splines was used for graphing smooth curves (see **Figure 1** and **Figure 2** in the **main text**). The time metric was CAP_D, which is a scaling of age based on corrected CAG. Site effects were ignored because the goal was description. Cubic splines can be computed with recursion formulas (De Boor, 2001). Five knots were used consisting of the minimum and maximum empirical values of CAP_D, and the quartiles. Suppose y_{jk} is the response for the jth person ($j = 1, \ldots, N$) at the kth time point ($k = 1, \ldots, n_j$). Let $f_h([CAP_D]_{jk})$ denote the hth basis function ($h = 1, \ldots, H - 1$). Then the cubic spline LMER model is

$$y_{jk} = \beta_0 + b_{0j} + \sum_{h=1}^{H-1} \beta_h f_h([CAP_D]_{jk}) + \epsilon_{jk}$$

3.4 Estimated Sample Size

Estimates of sample size required for a Phase II randomized controlled trial (RCT) of efficacy were based on power formulas for LMER (Yi and Panzarella, 2002; Heo and Leon, 2009). Phase II designs typically allow a higher Type I error rate than Phase III designs (Levy et al., 2006), and here we consider $\alpha = .10$ for a two-tailed test or $\alpha = .05$ for a one-tailed test. In an analysis not presented, the methods of Hu and Sale (Hu and Sale, 2003) indicated no evidence of informative dropout in the PREDICT-HD database. Therefore, all required sample size was

calculated under the assumption of non-informative dropout for the various scenarios discussed below. The endpoint was the individual variable rate of change (slope) over a two-year study with observations every six months (baseline, 6, 12, 28, 24), and the design was a two-arm Placebo (P) and Treatment (T) group study. The evaluation of efficacy was defined as the test of the null hypothesis that the rate of change in the P and T groups was equal. The null hypothesis can be evaluated based on parameter estimates in LMER.

Suppose y_{jk} is the outcome for the jth participant (j = 1, …, N) at the kth month (k = 1, …, n_j), and t denotes time in months. Assuming linear change over time, the LMER model can be written as the following,

$$y_{jk} = \beta_I + \beta_S t_{jk} + \beta_{\Delta I} g_j + \beta_{\Delta S} g_j t_{jk} + (b_{Ij} + b_{Sj} t_{jk} + \epsilon_{jk}) \qquad \text{(A2)}$$

where g is a dummy variable for group (g = 1 if the participant is in the T group, and 0 otherwise). In Equation A2, the βs are the fixed effects, with β_I being the P group intercept and β_S being the P group slope; $\beta_{\Delta I}$ is the difference in the intercepts among the groups, and $\beta_{\Delta S}$ is the difference among the slopes. The bs are random effects, and ϵ is random error. We make the typical assumptions, $[b_{Ij} \quad b_{Sj}]^T \sim \mathcal{N}(\mathbf{0}, \boldsymbol{G}) \perp \epsilon_{jk} \sim \mathcal{N}\left(0, \sigma_\epsilon^2 \boldsymbol{I}_j\right)$. The prime object of inference is $\beta_{\Delta S}$, as this is the difference in longitudinal change among the T and P groups. The null hypothesis of equality of T and P slopes is H_0: $\beta_{\Delta S} = 0$, and can be evaluated with a z-test. The Z statistic is Z = $\hat{\beta}_{\Delta S} / SE\left(\hat{\beta}_{\Delta S}\right)$ and leads to the rejection of H_0 when $|Z| > Z_{1-\alpha}$, with the latter being the $100(1 - \alpha)^{th}$ quantile of the standard normal distribution (single tailed).

The LMER model of A2 can be written more generally as

$$\boldsymbol{y}_j = \boldsymbol{X}_j \boldsymbol{\beta} + \boldsymbol{Z}_j \boldsymbol{b} + \boldsymbol{\epsilon}_j$$

where $\boldsymbol{X}$ is the design matrix of the fixed effects, $\boldsymbol{\beta}$ is the vector of fixed effects, $\boldsymbol{Z}$ is the design matrix of the random effects, $\boldsymbol{b}$ is the vector of random effects, and $\boldsymbol{\epsilon}$ is the vector of random errors. In this context, $\boldsymbol{X}_j$ for the jth person with no dropout has dimensions 5 × 4 with the first column being a vector of 1s, the second column a vector of time values (0, 6, 12, 18, 24), the third column a vector of g_j values, and the last column a vector of g_j × time values. The jth individual is defined as a dropout if their row dimension is less than 5.

The required sample size for the z-test depends on the variance of the responses,

$$\boldsymbol{V}_j = \boldsymbol{Z}_j \boldsymbol{G} \boldsymbol{Z}_j^T + \sigma_\epsilon^2 \boldsymbol{I}_j$$

V_j is 5 × 5 if the jth person has no dropout, but has reduced dimension under dropout similar to $\boldsymbol{X}_j$. Suppose α is the type I error rate (set to 0.05 for a one-tailed test, or .10 for two-tailed), and $1 - \gamma$ is power (set to 0.80). Then the required sample size for a single arm (half the total sample size) is

$$\frac{N}{2} = \frac{\left(Z_{1-\alpha} + Z_{1-\gamma}\right)^2 \left(\sum_l^2 \sum_m^L \boldsymbol{X}_{lm}^T \boldsymbol{V}_{lm}^{-1} \boldsymbol{X}_{lm} \pi_{lm}\right)_{4,4}^{-1}}{\beta_{\Delta S}^2} \qquad \text{(A3)}$$

where m refers to a specific dropout pattern, $\boldsymbol{X}_{lm}$ is the design matrix for the lth group with the mth dropout pattern, π_{lm} is the proportion of participants with the mth missing pattern in the lth group, and the π_{lm} sum to one among the dropout patterns within a group. The 4,4 subscript indicates the element in the 4th row and 4th column of the resulting matrix. When there is no

dropout, $\pi_{11} = \pi_{21} = 1$, and the right hand expression in the numerator is $(X_1^T V^{-1} X_1 + X_2^T V^{-1} X_2)^{-1}_{4,4}$, where the subscripts 1 and 2 represent the P and T groups, respectively.

Three non-informative dropout scenarios were considered: no dropout (0%), 10%, and 20%. The values pertain to the percentage of participants who dropped out in each group, not the total missing data. Dropout was identical for the P and T groups ($\pi_{1m} = \pi_{2m} = \pi_m$), and the dropout proportions, π_m, tapered to create a scenario in which dropout was slight very earlier in the hypothetical study, but increased over time. Five dropout patterns were considered, no dropout, missing last visit, missing last two visits, missing last three visits, and missing last four visits. **Supplementary Table 1** shows the π_m used for the three dropout conditions.

Supplementary Table 1. Dropout proportions (π) for computing required sample size

	**Dropout Condition**		
**Missing Visits**	**0%**	**10%**	**20%**
0	1.00	0.90	0.80
1	0	0.04	0.08
2	0	0.03	0.06
3	0	0.02	0.04
4	0	0.01	0.02

The variance components of Equation (A3) can be estimated using the PREDICT-HD database. However, PREDICT-HD is not a treatment study, and all gene-expanded participants are considered as members of the untreated placebo group in this context (not to be confused with gene-negative participants), with mean slope β_S. The object of inference, $\beta_{\Delta S}$, can be estimated as the proportion reduction of the placebo group slope under a hypothetical treatment, expressed as $\beta_{\Delta S} = \psi\beta_S$, where ψ is the proportion reduction in the placebo slope. Sample size was estimated for ψ = 0.20, 0.30, 0.40, 0.50, 0.60, 0.70. The last value, representing a 70% improvement by treatment, was the approximate percentage group slope difference over 12 weeks in an HD randomized clinical trial of tetrabenazine (Huntington Study Group, 2006), which is one of the few studies to show statistically significant efficacy (Armstrong et al., 2012). The range of effects is also consistent with clinical trials of other neurodegenerative diseases, such as multiple sclerosis (Altmann et al., 2009). **Table 4** in the **main text** presents parameter estimate for β_S, the unique elements of $\boldsymbol{G}$ (g_{11}, g_{12}, g_{22}), and σ_ϵ^2. These values can be used along with different design matrices, dropout proportions, etc., to provide estimates for a wide variety of clinical trial scenarios.

Typical for a Phase II RCT, we assumed a two-tailed statistical test with α = .10, 80% power, and a two-year study with measurements taken at baseline and every half-year, t = 0, 6, 12, 18, 24 months. The variance components were estimated using the PREDICT-HD participants in the Medium and High progression groups with age at entry from 30 to 50. Different dropout scenarios, including differential dropout by group, can be created by altering the design matrices of Equation (A3).

3.5 Check of Assumptions

LMER may not be appropriate for some variables in the analysis, such as TFC, due to extreme skewness in the marginal distribution very early in the disease (see **Figure 2** in the **main text**). Distributions for residuals and random effects were inspected for all variables. Though the distribution of the residuals and the site random effect was symmetric or nearly so for all variables, the distributions of the individual-level random effects were markedly negatively skewed for some variables, like TFC, and mildly positively skewed for other variables like TMS. It is acknowledged that alternative mixed models might be appropriate for such variables, like a cumulative logit model for the TFC, or a beta-binomial link model for the TMS. In additional analysis not presented, we found that the latter models provided similar inferences as LMER, and the decision was made to only present LMER results.

4. References

Altmann, D. R., Jasperse, B., Barkhof, F., Beckmann, K., Filippi, M., Kappos, L. D., Molyneux, P., Polman, C. H., Pozzilli, C., Thompson, A. J., Wagner, K., Yousry, T. A., and Miller, D. H. (2009). Sample sizes for brain atrophy outcomes in trials for secondary progressive multiple sclerosis. *Neurology* 72**,** 595–601. doi: 10.1212/01.wnl.0000335765.55346.fc.

Armstrong, M. J., Miyasaki, J. M., and American Academy Of, N. (2012). Evidence-based guideline: pharmacologic treatment of chorea in Huntington disease: report of the guideline development subcommittee of the American Academy of Neurology. *Neurology* 79**,** 597–603. doi: 10.1212/WNL.0b013e318263c443.

Avants, B. B., Epstein, C. L., Grossman, M., and Gee, J. C. (2008). Symmetric diffeomorphic image registration with cross-correlation: evaluating automated labeling of elderly and neurodegenerative brain. *Med. Image Anal.* 12**,** 26–41. doi: 10.1016/j.media.2007.06.004.

Aylward, E. H., Nopoulos, P. C., Ross, C. A., Langbehn, D. R., Pierson, R. K., Mills, J. A., Johnson, H. J., Magnotta, V. A., Juhl, A. R., Paulsen, J. S., and Predict-Hd Investigators and Coordinators of Huntington Study Group (2011). Longitudinal change in regional brain volumes in prodromal Huntington disease. *J. Neurol. Neurosurg. Psychiatry* 82**,** 405–410. doi: 10.1136/jnnp.2010.208264.

Beck, A., Steer, R., and Brown, G. (1993). *Beck Depression Inventory-II (BDI-II): Manual for Beck Depression Inventory-II.* Pearson.

Dale, A. M., Fischl, B., and Sereno, M. I. (1999). Cortical surface-based analysis. I. Segmentation and surface reconstruction. *Neuroimage* 9**,** 179–194. doi: 10.1006/nimg.1998.0395.

Dale, A. M., and Sereno, M. I. (1993). Improved Localizadon of Cortical Activity by Combining EEG and MEG with MRI Cortical Surface Reconstruction: A Linear Approach. *J. Cogn. Neurosci.* 5**,** 162–176. doi: 10.1162/jocn.1993.5.2.162.

De Boor, C. (2001). *A Practical guide to splines (Applied Mathematical Sciences).* Revised. Springer.

Derogatis, L. (1977). *Administration, scoring & procedures manual-I for the revised version and other instruments of the psychopathology rating scale series.* Baltimore, MD: John Hopkins University.

Doty, R. L., Shaman, P., Kimmelman, C. P., and Dann, M. S. (1984). University of Pennsylvania Smell Identification Test: a rapid quantitative olfactory function test for the clinic. *Laryngoscope* 94**,** 176–178.

Ekman, P., and Friesen, W. V. (1976). Measuring Facial Movement. *Environmental Psychology and Nonverbal Behavior* 1**,** 56–75. doi: Doi 10.1007/Bf01115465.

Farias, S. T., Mungas, D., Reed, B. R., Cahn-Weiner, D., Jagust, W., Baynes, K., and Decarli, C. (2008). The measurement of everyday cognition (ECog): scale development and psychometric properties. *Neuropsychology* 22**,** 531–544. doi: 10.1037/0894-4105.22.4.531.

Fischl, B., and Dale, A. M. (2000). Measuring the thickness of the human cerebral cortex from magnetic resonance images. *Proc. Natl. Acad. Sci. U. S. A.* 97**,** 11050–11055. doi: 10.1073/pnas.200033797.

Fischl, B., Liu, A., and Dale, A. M. (2001). Automated manifold surgery: constructing geometrically accurate and topologically correct models of the human cerebral cortex. *IEEE Trans. Med. Imaging* 20**,** 70–80. doi: 10.1109/42.906426.

Fischl, B., Salat, D. H., Busa, E., Albert, M., Dieterich, M., Haselgrove, C., Van Der Kouwe, A., Killiany, R., Kennedy, D., Klaveness, S., Montillo, A., Makris, N., Rosen, B., and Dale, A. M. (2002). Whole brain segmentation: automated labeling of neuroanatomical structures in the human brain. *Neuron* 33**,** 341–355.

Fischl, B., Salat, D. H., Van Der Kouwe, A. J., Makris, N., Segonne, F., Quinn, B. T., and Dale, A. M. (2004a). Sequence-independent segmentation of magnetic resonance images. *Neuroimage* 23 Suppl 1**,** S69–84. doi: 10.1016/j.neuroimage.2004.07.016.

Fischl, B., Van Der Kouwe, A., Destrieux, C., Halgren, E., Segonne, F., Salat, D. H., Busa, E., Seidman, L. J., Goldstein, J., Kennedy, D., Caviness, V., Makris, N., Rosen, B., and Dale, A. M. (2004b). Automatically parcellating the human cerebral cortex. *Cereb. Cortex* 14**,** 11–22.

Fischl, B., Sereno, M. I., and Dale, A. M. (1999a). Cortical surface-based analysis. II: Inflation, flattening, and a surface-based coordinate system. *Neuroimage* 9**,** 195–207. doi: 10.1006/nimg.1998.0396.

Fischl, B., Sereno, M. I., Tootell, R. B., and Dale, A. M. (1999b). High-resolution intersubject averaging and a coordinate system for the cortical surface. *Hum. Brain Mapp.* 8**,** 272–284.

Ghayoor, A., Vaidya, J. G., and Johnson, H. (2013). Development of a novel constellation based landmark detection algorithm. *Proc. SPIE Medical Imaging* 8669.

Golden, C. (1978). *Stroop Color and Word Test: Cat. No. 30150M; a Manual for Clinical and Experimental Uses.* Chicago, IL: Stoelting.

Grace, J., and Malloy, P. (2000). *Frontal systems behavior scale: professional manual.* Lutz, FL: Psychological Assessment Resources, Incorporated.

Han, X., Jovicich, J., Salat, D., Van Der Kouwe, A., Quinn, B., Czanner, S., Busa, E., Pacheco, J., Albert, M., Killiany, R., Maguire, P., Rosas, D., Makris, N., Dale, A., Dickerson, B., and Fischl, B. (2006). Reliability of MRI-derived measurements of human cerebral cortical thickness: the effects of field strength, scanner upgrade and manufacturer. *Neuroimage* 32**,** 180–194. doi: 10.1016/j.neuroimage.2006.02.051.

Heo, M., and Leon, A. C. (2009). Sample size requirements to detect an intervention by time interaction in longitudinal cluster randomized clinical trials. *Stat. Med.* 28**,** 1017–1027. doi: 10.1002/sim.3527.

Hu, C., and Sale, M. E. (2003). A joint model for nonlinear longitudinal data with informative dropout. *J. Pharmacokinet. Pharmacodyn.* 30**,** 83–103.

Huntington Study Group (1996). Unified Huntington's Disease Rating Scale: reliability and consistency. *Mov. Disord.* 11**,** 136–142. doi: 10.1002/mds.870110204.

Huntington Study Group (2006). Tetrabenazine as antichorea therapy in Huntington disease: a randomized controlled trial. *Neurology* 66**,** 366–372. doi: 10.1212/01.wnl.0000198586.85250.13.

Johnson, S. A., Stout, J. C., Solomon, A. C., Langbehn, D. R., Aylward, E. H., Cruce, C. B., Ross, C. A., Nance, M., Kayson, E., Julian-Baros, E., Hayden, M. R., Kieburtz, K., Guttman, M., Oakes, D., Shoulson, I., Beglinger, L., Duff, K., Penziner, E., Paulsen, J. S., and Predict-Hd Investigators of the Huntington Study Group (2007). Beyond disgust: impaired recognition of negative emotions prior to diagnosis in Huntington's disease. *Brain* 130**,** 1732–1744. doi: 10.1093/brain/awm107.

Jovicich, J., Czanner, S., Greve, D., Haley, E., Van Der Kouwe, A., Gollub, R., Kennedy, D., Schmitt, F., Brown, G., Macfall, J., Fischl, B., and Dale, A. (2006). Reliability in multi-

site structural MRI studies: effects of gradient non-linearity correction on phantom and human data. *Neuroimage* 30**,** 436–443. doi: 10.1016/j.neuroimage.2005.09.046.

Levy, G., Kaufmann, P., Buchsbaum, R., Montes, J., Barsdorf, A., Arbing, R., Battista, V., Zhou, X., Mitsumoto, H., Levin, B., and Thompson, J. L. (2006). A two-stage design for a phase II clinical trial of coenzyme Q10 in ALS. *Neurology* 66**,** 660–663. doi: 10.1212/01.wnl.0000201182.60750.66.

Lezak, M. D., Howieson, D., and Loring, D. (2004). *Neuropsychological assessment.* 4th ed. New York: Oxford University Press.

Long, J. D. (2012). *Longitudinal Data Analysis for the Behavioral Sciences Using R.* Sage Publications Inc.

O'Rourke, J. J., Beglinger, L. J., Smith, M. M., Mills, J., Moser, D. J., Rowe, K. C., Langbehn, D. R., Duff, K., Stout, J. C., Harrington, D. L., Carlozzi, N., and Paulsen, J. S. (2011). The Trail Making Test in prodromal Huntington disease: contributions of disease progression to test performance. *J. Clin. Exp. Neuropsychol.* 33**,** 567–579. doi: 10.1080/13803395.2010.541228.

Pierson, R., Johnson, H., Harris, G., Keefe, H., Paulsen, J. S., Andreasen, N. C., and Magnotta, V. A. (2011). Fully automated analysis using BRAINS: AutoWorkup. *Neuroimage* 54**,** 328–336. doi: 10.1016/j.neuroimage.2010.06.047.

Powell, S., Magnotta, V. A., Johnson, H., Jammalamadaka, V. K., Pierson, R., and Andreasen, N. C. (2008). Registration and machine learning-based automated segmentation of subcortical and cerebellar brain structures. *Neuroimage* 39**,** 238–247. doi: 10.1016/j.neuroimage.2007.05.063.

Reitan, R. (1958). Validity of the trail making test as an indicator of organic brain damage. *Percept. Mot. Skills* 8**,** 271–276.

Reuter, M., Rosas, H. D., and Fischl, B. (2010). Highly accurate inverse consistent registration: a robust approach. *Neuroimage* 53**,** 1181–1196. doi: 10.1016/j.neuroimage.2010.07.020.

Reuter, M., Schmansky, N. J., Rosas, H. D., and Fischl, B. (2012). Within-subject template estimation for unbiased longitudinal image analysis. *Neuroimage* 61**,** 1402–1418. doi: 10.1016/j.neuroimage.2012.02.084.

Rowe, K. C., Paulsen, J. S., Langbehn, D. R., Duff, K., Beglinger, L. J., Wang, C., O'Rourke, J. J., Stout, J. C., and Moser, D. J. (2010). Self-paced timing detects and tracks change in prodromal Huntington disease. *Neuropsychology* 24**,** 435–442. doi: 10.1037/a0018905.

Segonne, F., Dale, A. M., Busa, E., Glessner, M., Salat, D., Hahn, H. K., and Fischl, B. (2004). A hybrid approach to the skull stripping problem in MRI. *Neuroimage* 22**,** 1060–1075. doi: 10.1016/j.neuroimage.2004.03.032.

Segonne, F., Pacheco, J., and Fischl, B. (2007). Geometrically accurate topology-correction of cortical surfaces using nonseparating loops. *IEEE Trans. Med. Imaging* 26**,** 518–529. doi: 10.1109/TMI.2006.887364.

Sled, J. G., Zijdenbos, A. P., and Evans, A. C. (1998). A nonparametric method for automatic correction of intensity nonuniformity in MRI data. *IEEE Trans. Med. Imaging* 17**,** 87–97. doi: 10.1109/42.668698.

Smith, A. (1982). *Symbol Digit Modalities Test (SDMT) Manual (Revised).* Los Angeles, CA: Western Psychological Services.

Stroop, J. R. (1935). Studies of interference in serial verbal reactions. *J. Exp. Psychol.* 18**,** 643–662. doi: Doi 10.1037/0096-3445.121.1.15.

Verbeke, G., and Molenberghs, G. (2000). *Linear mixed models for longitudinal data.* New York: Springer.

Wechsler, D. (1981). *Manual for the Wechsler Adult Intelligence Scale - Revised.* New York, NY: Psychological Corporation.

World Health Organization (1988). *World Health Organization Psychiatric Disability Assessment Schedule (WHO/DAS), with a Guide to Its Use.* Geneva, Switzerland: World Health Organization.

Yi, Q., and Panzarella, T. (2002). Estimating sample size for tests on trends across repeated measurements with missing data based on the interaction term in a mixed model. *Control Clin. Trials* 23**,** 481–496.
